# NF-κB, iNOS, IL-6, and collagen 1 and 5 expression in healthy and keratoconus corneal fibroblasts after 0.1% riboflavin UV-A illumination

**DOI:** 10.1007/s00417-020-05058-z

**Published:** 2021-01-14

**Authors:** Tim Berger, Nóra Szentmáry, Lorenz Latta, Berthold Seitz, Tanja Stachon

**Affiliations:** 1 Department of Ophthalmology, Saarland University Medical Center, Kirrberger Str. 100, D-66424 Homburg, Saar Germany; 2 Dr. Rolf M. Schwiete Center for Limbal Stem Cell and Aniridia Research, Saarland University, Homburg, Saar Germany

**Keywords:** Keratoconus, Cross-linking, Corneal fibroblast, Inflammation, Collagen

## Abstract

**Purpose:**

To analyze the effect of riboflavin UV-A illumination on mRNA and protein expression of healthy (HCFs) and keratoconus human corneal fibroblasts (KC-HCFs), concerning the inflammatory markers NF-κB, iNOS, IL-6, and collagen 1 and 5 (Col 1/Col 5).

**Methods:**

Keratocytes were isolated from healthy (*n* = 3) and keratoconus (KC) corneas (*n* = 3) and were cultivated in basal medium with 5% fetal calf serum, which resulted in their transformation into human corneal fibroblasts (HCFs/KC-HCFs). Cells underwent 0.1% riboflavin UV-A illumination for 250 s (CXL). NF-κB, iNOS, IL-6, Col 1, and Col 5 expression was investigated by qPCR and Western blot analysis. IL-6 concentration of the cell culture supernatant and cell lysate was determined by ELISA.

**Results:**

In untreated KC-HCFs, NF-κB (*p* = 0.0002), iNOS (*p* = 0.0019), Col 1 (*p* = 0.0286), and Col 5 (*p* = 0.0054) mRNA expression was higher and IL-6 expression was lower (*p* = 0.0057), than in healthy controls. In HCFs, CXL led to an increased NF-κB (*p* = 0.0286) and IL-6 (*p* = 0.0057) mRNA expression. The IL-6 concentration in the cell culture supernatant was increased in HCFs (*p* = 0.0485) and KC-HCFs (*p* = 0.0485) after CXL. CXL increased intracellular IL-6 concentration only in KC-HCFs (*p* = 0.0357). In the HCF group (*p* = 0.0286), an increased Col 1 mRNA expression after CXL could be observed.

**Conclusion:**

Our study confirmed altered gene expression in untreated KC-HCFs compared to untreated HCFs. Riboflavin UV-A illumination affected gene expression only in HCFs. Increased IL-6 concentration in the cell culture supernatant and cell lysate indicate a secondary inflammatory response of HCFs and KC-HCFs to riboflavin UV-A illumination.

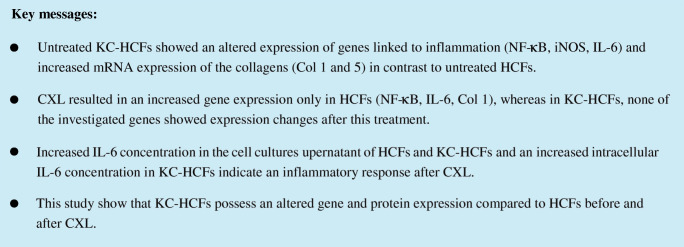



## Introduction

Keratoconus (KC) is a bilateral, progressive ectatic corneal disease, which results in a cone-shaped protrusion of the corneal tissue due to the thinning of the corneal stroma. These morphological changes are associated with reduced visual acuity, progressive myopia, and irregular astigmatism. As part of the progressive stromal thinning in advanced KC, the rupture of Descemet’s membrane may cause an acute hydrops. This stromal edema subsequently results in corneal scarring, which makes penetrating keratoplasty in most cases necessary in order to improve visual acuity [1].

In general, an annual incidence of 2/100,000 and a prevalence of 54.5/100,000 is assumed [2]. These data are based on a long-term study from 1935 to 1982 and may be outdated due to progress in diagnostics. A recent study indicates that the annual incidence (13.3/100,000) and the prevalence (265/100,000) could be 5-fold higher than previously reported [3]. Although KC is known to affect all ethnic groups, its development shows geographical variability with a higher incidence among the Asian population [4].

The etiology of KC has not been clarified so far, but a complex, multifactorial pathogenesis is assumed. In most cases, KC occurs as an isolated disease without any evidence of underlying concomitant diseases. KC shows a higher familial incidence, which could be an indicator of a genetic predisposition. Biochemical alterations associated with increased proteolytic activity are discussed to be responsible for the degradation of corneal structural components resulting in the biomechanical weakening of the cornea. It is also assumed that a different distribution and lower number of stromal lamellae in KC is associated with reduced corneal rigidity and thinning, compared to normal corneas. Besides, KC is more frequent in Down syndrome, Leber’s congenital amaurosis, Ehlers-Danlos syndrome, and osteogenesis imperfecta [5].

Although KC is classified in the common literature as a non-inflammatory disease, there are data referring to potential underlying inflammatory components. In a previous study of our research group, increased nuclear factor kappa B p65 (NF-κB) mRNA and protein expression, and an increased inducible NO synthase (iNOS) mRNA expression could be verified in human keratoconus fibroblasts (KC-HCFs), compared to healthy controls, which refers to corneal inflammation in KC [6]. In addition, an increased interleukin-6 (IL-6), tumor necrosis factor-α (TNF-α), and matrix metalloprotease-9 (MMP-9) concentration could be measured in tear fluid of KC patients, and a higher concentration of these proteins was associated with a more severe form of KC [7]. Besides, several studies have demonstrated increased oxidative stress and altered metabolism in KC stromal cells [8, 9].

The healthy corneal stroma is rich in collagen 1A1 (Col 1) and contains a relatively large amount of type 5A1 collagen (Col 5). Corneal collagen fibrils consist of type I collagen molecules that are integrated into heterotypic fibrils together with type 5 collagen. These interactions ensure the proper organization of the collagen lamellae and regulate the diameter of the collagen fibrils to maintain corneal transparency [10]. It has been shown that KC is associated with altered collagen fibrillar diameter, alterations in collagen distribution, and changes in fibril orientation within the corneal stroma [11, 12].

Conservative and surgical KC treatment options depend on the progression of the disease. In the early stages, refractive changes can be corrected using spectacles or contact lenses. Minimally invasive surgical treatment options include corneal collagen cross-linking (CXL) and the implantation of intracorneal ring segments, using these two surgical treatment options either separately or in combination. In advanced stages, either lamellar or penetrating keratoplasty could be necessary [13].

The aim of CXL is to increase the stiffness of the corneal tissue by forming further bonds between the collagen fibrils. Therefore, the simultaneous use of riboflavin (vitamin B_2_) as a photosensitizer and a 370-nm wavelength UV-A light leads to cross-links between the collagen fibrils due to a photochemical reaction, resulting in stabilization of the current state [14].

Riboflavin is excited by absorbing energy of the UV-A light (singlet riboflavin), and it is converted into a triplet state. Type I and type II reactions can be distinguished. For the type II reaction, oxygen is required to form singlet oxygen, an oxygen radical, which react with the carbonyl group of the collagen [14]. The type I reaction takes place after that the oxygen is exhausted, in which the excited riboflavin (triplet state) is transferring a hydrogen atom or electron to biomolecules such as lipids, proteins, and nucleic acids or generates reactive oxygen species (ROS) like superoxide anion, hydroxyl radical, or hydrogen peroxide [15]. As a consequence of these reactions, the collagen fibrils are cross-linked, and thus, the current stage of the disease is stabilized.

The first clinical results of CXL were published in 2003 by Wollensak et al. to conclude that the progression of KC could be slowed down [16].

Nowadays, CXL represents an important therapeutic option for KC patients, but the cellular effects are not well understood. In a previous study, we have shown that riboflavin UV-A illumination is associated with increased apoptosis and decreased viability of keratoconus stromal cells [17].

In the present work, our purpose was to analyze the effects of riboflavin UV-A illumination on mRNA and protein expression of healthy (HCFs) and keratoconus human corneal fibroblasts (KC-HCFs), concerning the inflammatory markers NF-κB, iNOS, IL-6, and collagen 1 and 5 (Col 1/Col 5). As a first step, our purpose was to investigate NF-κB, iNOS, IL-6, and Col 1/Col 5 mRNA and protein expression differences between untreated healthy and KC human corneal fibroblasts. As a second step, we aimed to investigate the inflammatory response (NF-κB, iNOS, IL-6) related to the increased free oxygen species formation after riboflavin UV-A illumination in these cells. Thirdly, we aimed to examine whether a riboflavin UV-A illumination has an impact on Col 1/Col 5 expression.

## Materials and methods

### Ethics approval and consent to participate

This study was performed in accordance with the Declaration of Helsinki and was approved by the Ethics Committee of Saarland/Germany (No. 41/18). An informed consent was obtained from all participants with KC, before keratoplasty was performed.

### Cell culture

Three normal human corneas were obtained from the LIONS Cornea Bank Saar-Lor-Lux, Trier/Westpfalz. Donor corneoscleral buttons, which did not match the criteria for transplantation (less than 1800 endothelial cells/mm^2^), have been used for the experiments. In addition, central corneal buttons with a diameter of 8.0 mm were obtained from elective penetrating keratoplasties of 3 KC patients who did not undergo previous ocular surgery. Immediately after elective penetrating keratoplasty, the explanted corneal buttons were further processed for cell cultivation. The donor corneoscleral buttons were explanted within 24 h postmortem. As these corneas were not considered suitable for elective keratoplasty, they were released for study purposes. Therefore, three healthy human corneas and three corneas from KC patients were used for cell cultivation. Further information on donor corneoscleral buttons and KC patients is provided in Tables 1 and 2.

**Table 1:** Descriptive data of donor corneoscleral buttons

	Donor age (years)	Gender	Side	Cause of death	Exclusion from corneal transplantation
HCF 1	81	Female	Right	Intracranial hemorrhage	< 1800 endothelial cells/mm^2^
HCF 2	90	Male	Right	Bronchial carcinoma	Used for Descemet membrane endothelial keratoplasty tissue preparation
HCF 3	69	Male	Left	Intracranial hemorrhage	< 1800 endothelial cells/mm^2^

**Table 2:** Keratoconus patient characteristics corresponding to the primary cell cultures

	Patient age (years)	Gender	Side	Keratoconus grading (ABCD system, Belin)	Co-morbidities	Corneal explant size (mm)	Time from explantation to cell cultivation (h)
KC-HCF 1	51	Male	Right	A4/B4/C4/D4+	Arterial hypertension	8.0	< 12
KC-HCF 2	31	Male	Left	A4/B4/C4/D4+	Healthy	8.0	< 12
KC-HCF 3	36	Female	Right	A0/B2/C4/D2+	Healthy	8.0	< 12

Cell culture work was performed under sterile conditions. The donor corneoscleral buttons were first rinsed in phosphate-buffered saline (PBS) (Sigma-Aldrich, St. Louis, USA); then, adjacent scleral parts with 2 mm of the clear cornea were removed, and the central corneal button was cut into pieces using a disposable surgical scalpel. For isolation of the keratocytes, the tissue was incubated with 1.0 mg/ml collagenase A (Hoffmann-La Roche, Basel, Switzerland) together with cell culture medium consisting of Dulbecco’s modified Eagle’s medium (DMEM/F12) (Thermo Fisher Scientific, Waltham, MA, USA), 5% fetal calf serum (FCS) (Thermo Fisher Scientific, Waltham, MA, USA), and 1% penicillin-streptomycin (P/S) (Sigma-Aldrich, St. Louis, USA) for 24 h at 37 °C, which is a common standard procedure for fibroblast cell culture work. The above-mentioned mixture (DMEM/F12, FCS, P/S) is defined in the following text as cell culture medium. The digested tissue and cells were centrifuged at 800*g* for 7 min, and the supernatant was discarded. After resuspending the cell pellet with 1 ml PBS, the cell suspension was seeded in a 75-cm^2^ cell culture flask containing 13 ml of cell culture medium, which was changed every third or fourth day until the cells reached confluence. Cultivation was performed in an incubator at 37 °C with 95% relative humidity and 5% CO_2_ atmosphere. The confluent cell culture was harvested with trypsin EDTA (Sigma-Aldrich, St. Louis, USA) and was split into several 75-cm^2^ cell culture flasks. The influence of the FCS resulted in a differentiation of the keratocytes into fibroblasts. Therefore, we specify them further on as “human corneal fibroblasts” (HCFs) and “keratoconus human corneal fibroblasts” (KC-HCFs). Experiments were performed using passages three to eight of the cells.

## Riboflavin UV-A illumination of HCFs and KC-HCFs

The cell culture medium was changed 24 h before starting the experiments. Riboflavin (Sigma-Aldrich, St. Louis, USA) was diluted with cell culture medium to a concentration of 0.1%, was protected from light, and was stored at 4 °C for the experiments (further on “riboflavin solution”).

As a first step, the cell culture supernatant was removed, and either 5 ml cell culture medium or 5 ml of the riboflavin solution was pipetted into the cell culture flask under the sterile bench. Thereafter, the cell culture was quickly placed in the dark or was illuminated with 375-nm UV-A light for 250 s (2 J/cm^2^) in an illumination box. After the immediate removal of the cell culture medium or the riboflavin solution, the flask was rinsed twice with 10 ml PBS. We added to each cell culture 10 ml cell culture medium, and cells were cultured for 24 h or 48 h at 37 °C before measurements. In order to determine the IL-6 concentration in the cell culture supernatant, 1.5 ml cell culture supernatant was collected from each flask before harvesting of the cells (24 h or 48 h after the experiment) and was stored at − 80 °C.

Then, the cells were harvested with trypsin EDTA and were stored at − 80 °C until further use.

### RNA isolation and cDNA synthesis

RNA was isolated using the RNA Purification Plus Micro Kit (Norgen Biotek Corp., Thorold, Canada), following the instructions provided by the manufacturer. RNA quantity was determined using a UV/VIS spectrophotometer (Analytik Jena AG, Jena, Germany), and the eluted RNA was stored at − 80 °C for further use.

The cDNA synthesis was performed using the OneTaq RT-PCR Kit (New England BioLabs, Frankfurt a. M., Germany) according to the instructions provided for the Kit. For cDNA synthesis, 1 μg of total RNA was used as template for all samples. The synthesized cDNA was stored at − 20 °C for further use.

### Quantitative PCR

The reaction mix (total volume: 9 μl) for quantitative PCR (qPCR) consisted of 1 μl of the specific primer solution, 5 μl SYBR Green Mix (Qiagen N.V., Venlo, Netherlands), and 3 μl nuclease-free water. The qPCR reactions were performed using the QuantStudio 5 real-time PCR system (Thermo Fisher Scientific, Waltham, MA, USA). Samples were run in 9 μl volume using 1.5 μl cDNA according to the manufacturer’s instructions. The amplification conditions (40 cycles) were 95 °C for 10 s, 60 °C for 30 s, and 95 °C for 15 s. All samples were measured in duplicate. Values were normalized to Tata-binding protein (TBP) expression levels as endogenous control gene, using the ∆∆CT method. The fold change (2^∆∆CT-value^) was used for the statistical analysis. A list of primers used for qPCR is summarized in Table 3. Normal HCFs (incubated in the dark with cell culture medium for 250 s) were used as controls (fold change = 1). Gene expression was measured for predetermined time points (Table 4), which have been chosen according to previous measurement series of our research group [18].

**Table 3:** Primer pairs used for qPCR

Primer	Primer sequence or QIAGEN catalog number	Manufacturer (company, city, country)
Collagen 1A1 (Col 1)	QT00037793	Qiagen N.V., Venlo, Netherlands
Collagen 5A1 (Col 5)	QT00044527	Qiagen N.V., Venlo, Netherlands
Inducible NO synthase (iNOS)	CTGGCAAGCCCAAGGTCTAT GGAGGCTCCGATCAATCCAG	Eurofins Genomics Germany GmbH, Ebersberg, Germany
Interleukin-6 (IL-6)	QT00083720	Qiagen N.V., Venlo, Netherlands
Nuclear factor kappa B p65 (NF-κB)	QT02324308	Qiagen N.V., Venlo, Netherlands
Tata-binding protein (TBP)	QT00000721	Qiagen N.V., Venlo, Netherlands

**Table 4:** The examined gene expressions and the respective measurement time points, following riboflavin UV-A illumination

Gen	Time points of gene expression measurement after riboflavin UV-A illumination
Inducible NO synthase (iNOS)	48 h
Interleukin-6 (IL-6)	24 h + 48 h
Collagen 1A1 (Col 1)	24 h
Collagen 5A1 (Col 5)	24 h
Nuclear factor kappa B p65 (NF-κB)	24 h
Tata-binding protein (TBP)	24 h + 48 h

### Protein quantification and Western blot analysis

Cells were lysed in RIPA buffer (Thermo Fisher Scientific, Waltham, MA, USA) 48 h after the experiments, and protein concentration was determined using the Pierce™ BCA Protein Assay Kit (Thermo Fisher Scientific, Waltham, MA, USA). The measurement was performed using the Tecan Infinite F50 Absorbance Microplate Reader (Tecan Group AG, Männedorf, Switzerland) with 560-nm wavelength. Bovine serum albumin (BSA) was used as a standard. The measurements were performed in duplicate. For Western blot analysis, samples with 20 μg total protein were boiled in a sample buffer for 5 min at 95 °C and were loaded on a precast 4–12% NuPage™ Bis-Tris SDS Gel (Invitrogen, Waltham, MA, USA). The first well was loaded with 3 μl Precision Plus Protein™ Dual Color Standard (Bio-Rad Laboratories, Hercules, USA) to determine the molecular weight. NuPAGE™ MOPS SDS Running Buffer (20×) (Thermo Fisher Scientific, Waltham, MA, USA) was used as an electrophoresis buffer. After protein separation, semi-dry blotting on a nitrocellulose membrane was performed using the Trans-Blot Turbo Transfer System (Bio-Rad Laboratories, Hercules, USA), according to the pre-installed blotting protocol for high molecular weight proteins. The membrane was washed three times with 15 ml Western Froxx washing solution (BioFroxx GmbH, Einhausen, Germany) for 5 min, which was followed by primary antibody incubation at 4 °C. A list of antibodies used for Western blot analysis is summarized in Table 5. The primary antibodies were diluted in a combined blocking and secondary antibody solution (WesternFroxx anti-Mouse HRP/anti-Rabbit HRP; BioFroxx GmbH, Einhausen, Germany). Calnexin was used as a reference protein. After the removal of the antibody solution, the membrane was incubated with 15 ml washing solution three times. Protein band detection was performed with the Western Lightning Plus Chemiluminescence Reagent (PerkinElmer Inc., Waltham, MA, USA). The chemiluminescence signal was detected using the ImageQuant LAS 400 (GE Healthcare, Chicago, USA). Thereafter, the membrane stripping was performed using the Western Froxx stripping solution (BioFroxx GmbH, Einhausen, Germany).

**Table 5:** Catalog number, manufacturer/distributor, and dilution of antibodies used for Western blot analysis

Antibody	Catalog number	Manufacturer (company, city, country)	Dilution
Calnexin (anti-rabbit)	ADI-SPA-865	Enzo Life Sciences GmbH, Lörrach, Germany	1:1000
Collagen 1A1 (anti-rabbit)	84336	Cell Signaling Technology, MA, USA	1:1000
Collagen 5A1 (anti-rabbit)	ab7046	Abcam, Cambridge, UK	1:5000
Inducible NO synthase (anti-rabbit)	ab3523	Abcam, Cambridge, UK	1:1000
Nuclear factor kappa B p65 (anti-rabbit)	8242	Cell Signaling Technology, MA, USA	1:1000

### ELISA

Enzyme-linked immunosorbent assay (ELISA) was used to determine the IL-6 concentration of the cell culture supernatant (24 h and 48 h after experiments) and cell lysate (48 h after experiments).

The detection procedure was based on the sandwich ELISA technique’s principle using the Human IL-6 Quantikine ELISA Kit (R&D Systems, Minneapolis, USA), according to the provided instructions, at room temperature. Measurements were performed in duplicate with 100 μl of the antigen-containing sample solution (cell culture supernatant or lysate). The quantitative detection of the IL-6 concentration was measured by a Tecan Infinite F50 Absorbance Microplate Reader using a standard provided curve. Measured IL-6 concentrations were divided by the total protein concentrations to obtain the IL-6 concentration in picogram per milligram protein. The quotient (pg IL-6/mg protein) was used for further statistical analysis.

### Statistical analysis

GraphPad Prism 7.04 was used for the statistical analysis and for creating the diagrams. All data were expressed as mean ± SEM. Difference between groups was determined using Mann-Whitney *U* test. *p* values < 0.05 were considered statistically significant.

## Results

### qPCR

#### Gene expression in untreated HCFs and KC-HCFs

**Fig. 1:**
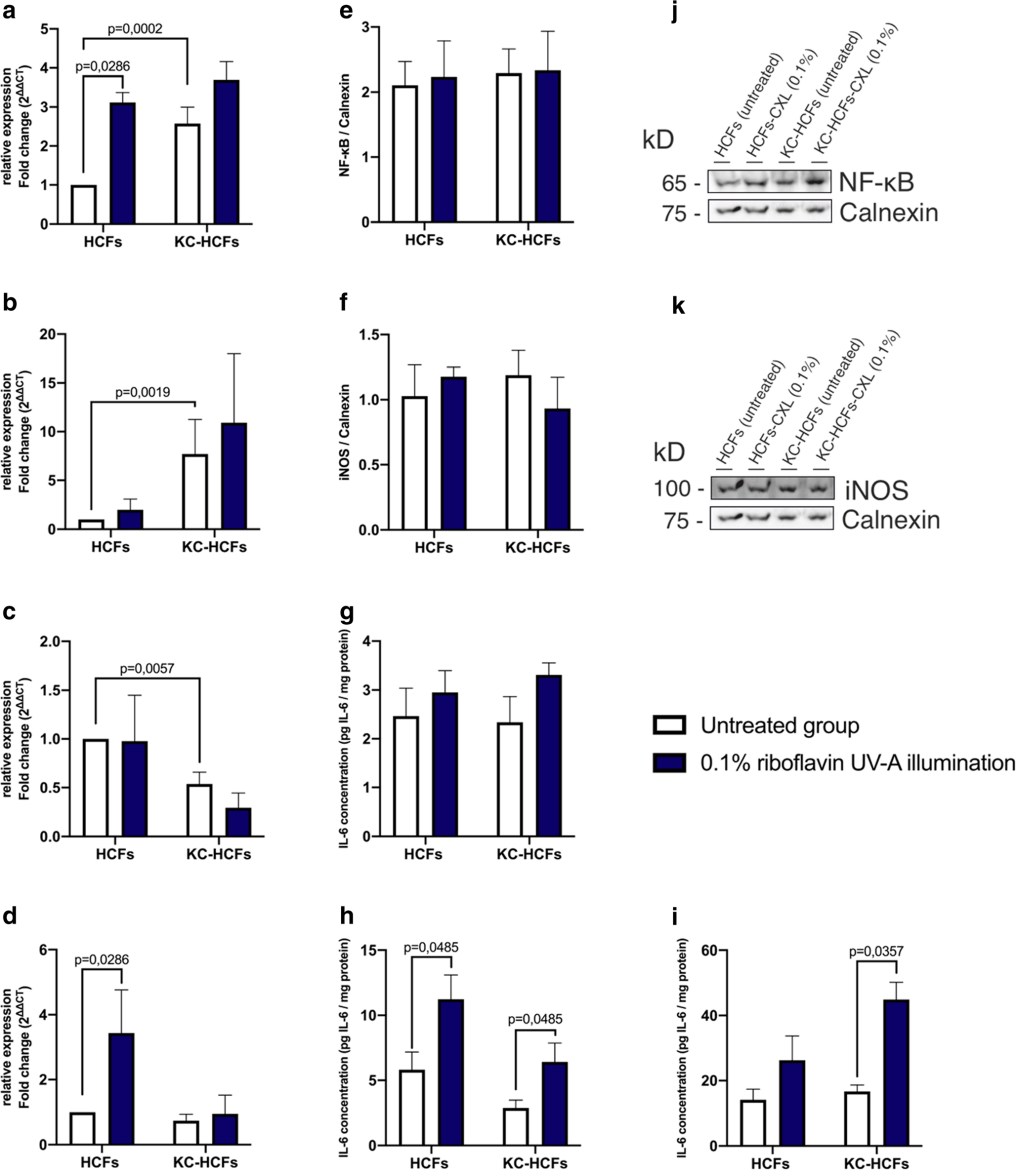
Effect of riboflavin UV-A illumination on NF-κB, iNOS, and IL-6 mRNA and protein expression (untreated group: white bars; riboflavin UV-A illumination group: blue bars). NF-κB (24 h), iNOS (48 h), and IL-6 (24 h and 48 h) gene expression was measured 24 or 48 h after riboflavin UV-A illumination. NF-κB and iNOS protein expression analysis was performed 48 h after riboflavin UV-A illumination. IL-6 concentration of the cell culture supernatant (24 h and 48 h after treatment) and cell lysate (48 h after treatment) was determined by ELISA. (A–D) NF-κB, iNOS, and IL-6 quantitative mRNA analysis. Data show mean ± SEM of at least 3 independent experiments in duplicate. (E, F) Relative quantification of NF-κB and iNOS Western blot analysis. Calnexin was used as loading control and to calculate the relative protein expression levels. Data show mean ± SEM of at least 3 independent experiments in duplicate. (G–I) IL-6 concentration was determined in cell culture supernatant and cell lysate 24 and/or 48 h after riboflavin UV-A illumination. Measured IL-6 concentrations were divided by the total protein concentrations, to obtain the IL-6 concentration in pg per mg protein, these values are displayed (pg IL-6/mg protein). Data show mean ± SEM of at least 3 independent experiments in duplicate. (J, K) Representative NF-κB and iNOS Western blots. Significant *p* values (< 0.05) are highlighted in the diagrams

**Fig. 2:**
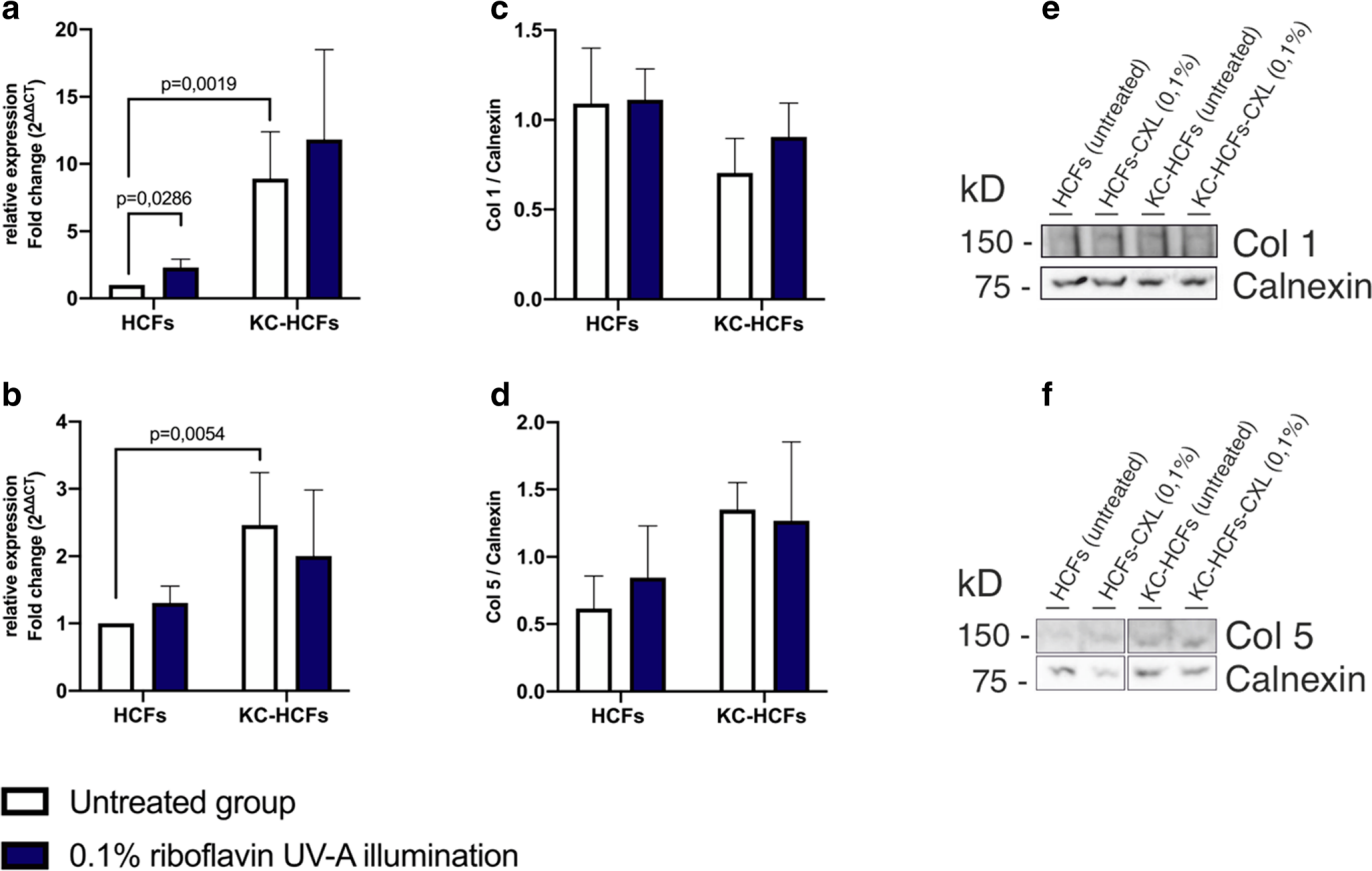
Effect of riboflavin UV-A illumination on Col 1 and Col 5 mRNA and protein expression (untreated group: white bars; riboflavin UV-A illumination group: blue bars). Col 1 and Col 5 mRNA expression was measured 24 h after riboflavin UV-A illumination. The protein expression analysis was performed 48 h after treatment. (A, B) Col 1 and Col 5 quantitative mRNA analysis. Data show mean ± SEM of at least 3 independent experiments in duplicate. (C, D) Relative quantification of Col 1 and Col 5 Western blot analysis. Calnexin was used as loading control and to calculate the relative protein expression levels. (E, F) Representative Col 1 and Col 5 Western blots. Significant *p* values (< 0.05) were highlighted in the diagrams

NF-κB and iNOS mRNA expression was higher (*p* = 0.0002; *p* = 0.0019) and IL-6 (*p* = 0.0057) mRNA expression was lower in untreated KC-HCFs than in untreated healthy controls (Fig. 1). Col 1 and Col 5 mRNA expression was higher in KC-HCFs than in HCFs (*p* = 0.0019; *p* = 0.0054) (Fig. 2).

#### Gene expression in HCFs and KC-HCFs after riboflavin UV-A illumination

NF-κB and IL-6 mRNA expression increased (*p* = 0.0286; *p* = 0.0286) in HCFs after 0.1% riboflavin UV-A illumination. NF-κB and iNOS mRNA expression remained unchanged in KC-HCFs after treatment (*p* = 0.2788; *p* = 0.7457) and iNOS mRNA expression did not change through 0.1% riboflavin UV-A illumination in HCFs or KC-HCFs (*p* = 0.3143) (Fig. 1).

Col 1 mRNA expression increased in HCFs following 0.1% riboflavin UV-A illumination (*p* = 0.0286) and Col 5 expression remained unchanged in HCFs and KC-HCFs after treatment (*p* = 0.0971; *p* = 0.4623) (Fig. 2).

### Western blot

NF-κB (*p* = 0.7308), iNOS (*p* = 0.6282), Col 1 (*p* = 0.3660), and Col 5 (*p* = 0.1255) protein expression did not differ between untreated HCFs and KC-HCFs. NF-κB (*p* = 0.9999; *p* = 0.9999), iNOS (*p* = 0.9999; *p* = 0.6667), Col 1 (*p* = 0.3660; *p* = 0.5167), and Col 5 (*p* = 0.8571; *p* = 0.5714) protein expression remained unchanged in HCFs or KC-HCFs through 0.1% riboflavin UV-A illumination (Figs. 1 and 2).

### ELISA

IL-6 concentration in the cell culture supernatant did not differ between untreated HCFs and KC-HCFs (24 h: *p* = 0.8785; 48 h: *p* = 0.0650). Twenty-four hours after treatment, IL-6 concentration in the cell culture supernatant did not change in HCFs and KC-HCFs (*p* = 0.4970; *p* = 0.1333). Nevertheless, 48 h after 0.1% riboflavin UV-A illumination, IL-6 concentration in the cell culture supernatant increased in HCFs (*p* = 0.0485) and KC-HCFs (*p* = 0.0485), compared to baseline values (Fig. 1).

In the cell lysate, IL-6 concentration did not differ between untreated HCFs and KC-HCFs (*p* = 0.7922). Nevertheless, in the cell lysate, IL-6 concentration increased in KC-HCFs 48 h after 0.1% riboflavin UV-A illumination (*p* = 0.0357). In HCFs, IL-6 concentration in the cell lysate remained unchanged after treatment (*p* = 0.1667) (Fig. 1).

## Discussion

Little is known about the pathogenesis of KC, but along the reported changes in collagen metabolism [11, 12], the suspicion of a chronic inflammatory component is increasingly coming to the fore. Since cross-linking was first introduced by Wollensak et al. in 2003 [16], it became an indispensable therapeutic option for treating ectatic corneal disorders. However, the cell biological effects after CXL were barely investigated. Therefore, in this study, we treated corneal fibroblasts in vitro with riboflavin UV-A illumination, to examine the impact of CXL regarding expression changes of inflammatory markers NF-κB, iNOS, IL-6, and collagen 1 and 5 in HCFs and KC-HCFs.

We could demonstrate changes in gene expression linked to inflammation in untreated KC-HCFs. NF-κB is an important modulator of many cell biological pathways through its influence on more than 500 genes, mainly genes associated with inflammatory responses [19]. Both iNOS and IL-6 are influenced by NF-κB [20, 21]. Additionally, iNOS is activated by IL-1, TNF-α, and interferon-γ and leads to the production of nitric oxygen as part of inflammatory processes [22]. Besides elevated inflammatory markers in the tear fluid of KC patients [7], Karamichos et al. reported an increase in metabolites associated with oxidative stress in KC keratocytes, which could be related to a chronic inflammatory condition [8]. We argue that increased iNOS mRNA expression in KC-HCFs is due to underlying inflammatory processes in KC, which may be induced by increased production of ROS and other metabolic pathway changes. In another study, KC-HCFs showed an increased ROS production, through which these cells got more vulnerable to additional oxidative stress than HCFs [23]. As CXL itself results in oxidative stress [14], resulting in a secondary inflammatory reaction, we investigated its effects on HCFs and KC-HCFs. In a previous study of our research group, we demonstrated an increased apoptotic rate of stromal cells and reduced cell viability in KC-HCFs, after riboflavin UV-A illumination [17], whereas in another study, normal HCFs were less vulnerable [24]. Increased keratocyte apoptosis after CXL could also be demonstrated in KC corneas [25]. Myofibroblastic transformation following CXL also differed significantly between KC and normal corneal stromal cells [17, 24].

In our present study, we could demonstrate that riboflavin UV-A illumination is associated with a significant increase in NF-κB and IL-6 mRNA expression in HCFs, but in KC-HCFs, no changes were detected. This could be related to an altered NF-κB and IL-6 mRNA expression in untreated KC-HCFs compared to untreated HCFs and may refer to an underlying cellular stress reaction in KC-HCFs [8], with an insufficient capacity of KC-HCFs to further increase gene expression, due to possibly restricted regulatory mechanisms. In a recently published study of our research group, we investigated the influence of hypoxic stress on HCFs and KC-HCF [18]. Interestingly, increased gene expression of inflammatory markers (NF-κB and iNOS) was only observed within the HCF group, whereas in KC-HCFs, gene expression was not influenced through hypoxia. Increased ROS production after riboflavin UV-A illumination, as well as hypoxia, could result in inflammatory reactions, but further investigation is necessary to clarify why changes in gene expression could not be measured in KC-HCFs after those specific stress stimuli.

Although no IL-6 mRNA expression changes in KC-HCFs could be measured after riboflavin UV-A illumination, IL-6 protein amount increased significantly in the cell culture supernatant through riboflavin UV-A illumination in both HCFs and KC-HCFs. However, a difference in the IL-6 concentration of the cell culture supernatant could only be observed after 48 h, indicating that this might be a delayed reaction of the corneal fibroblasts after riboflavin UV-A illumination, which should be considered in future studies. Interestingly, riboflavin UV-A illumination resulted in an increased intracellular IL-6 concentration only in KC-HCFs, which was not accompanied by altered mRNA expression. This could indicate that KC-HCFs can only partially compensate the induced stress stimulus and therefore have a significant intracellular stress response.

In this study, corneas with advanced KC stages (Belin KC classification) and corneal scarring were used [26]. Most interestingly, we detected an increased Col 1 and Col 5 mRNA expression in KC-HCFs compared to HCFs, which refers to the disturbance of the collagen metabolism in KC corneas [11, 12]. In addition, the Col 1 and Col 5 mRNA expression values have shown a larger variability in KC-HCFs than in HCFs. These variable values were confirmed through repeat measurements. We suggest that these differences depend on the individual patient’s cornea and in addition, during KC progression, gene expression could also change. It is important to investigate these results in future studies, for example, by comparing the mRNA and protein expression of KC corneas with or without scars or under consideration of the KC stage.

Furthermore, we investigated whether CXL is associated with changes in collagen expression. In this study, we observed increased Col 1 mRNA expression only in HCFs without significant protein expression changes in HCFs or KC-HCFs following riboflavin UV-A illumination. Another study described a significant Col 1 and Col 5 protein expression decrease after 0.1% riboflavin UV-A illumination in KC-HCFs, but not in HCFs, nevertheless, using 3D cell cultures [27]. To which extent these changes could be of cell biological or clinical relevance must be critically analyzed.

Finally, it is necessary to mention certain limitations of our study concerning the interpretation of the results. On the one hand, the avascular stroma represents a nutrient-poor tissue. Therefore, in vitro experiments are not equally applicable to in vivo models. Our isolated keratocytes dedifferentiated into fibroblasts during cell cultivation, using FCS, which could probably have a significant impact on gene and protein expression. The results of the 2D culture are also not directly transferable to the in vivo model, but give a good indication for further studies. In addition, there is no suitable in vivo model or animal model to study KC. In summary, we found expression differences between untreated and treated corneal fibroblasts, which is an important input for future studies.

## Conclusions

In this study, altered mRNA expression of the inflammatory markers (NF-κB, iNOS, IL-6), as well as the collagens (Col 1, Col 5), could be detected in untreated KC-HCFs. Riboflavin UV-A illumination resulted in significant gene expression changes only in HCFs. Increased IL-6 concentrations in the cell culture supernatant (HCFs and KC-HCFs) and cell lysate (KC-HCFs) indicated an inflammatory response after this treatment. The effects of cross-linking on gene and protein expression have rarely been investigated, and therefore, the results of this study provide a basis for further research projects.
